# Disordered Eating Behaviors and Food Addiction among Nutrition Major College Students

**DOI:** 10.3390/nu8110673

**Published:** 2016-10-26

**Authors:** Zhiping Yu, Michael Tan

**Affiliations:** 1 Department of Nutrition and Dietetics, University of North Florida, Jacksonville, FL 32224, USA; 2 Department of Nutrition and Food Sciences, University of Rhode Island, Kingston, RI 02881, USA; MichaelATan@gmail.com

**Keywords:** eating disorder, disordered eating behaviors, food addiction, nutrition students

## Abstract

Evidence of whether nutrition students are free from food-related issues or at higher risk for eating disorders is inconsistent. This study aimed to assess disordered eating behaviors and food addiction among nutrition and non-nutrition major college students. Students (*n* = 967, ages 18–25, female 72.7%, white 74.8%) enrolled at a public university completed online demographic characteristics surveys and validated questionnaires measuring specific disordered eating behaviors. Academic major category differences were compared. Additionally, high risk participants were assessed by weight status and academic year. Overall, 10% of respondents were a high level of concern for developing eating disorders. About 10.3% of respondents met criteria for food addiction. In addition, 4.5% of respondents had co-occurrence of eating disorder risk and food addiction risk out of total respondents. There were no significant differences in level of concern for developing an eating disorder, eating subscales, or food addiction among academic majors. The percentage of high risk participants was lower in the underweight/normal weight group than in the overweight/obese group in health-related non-nutrition major students but not in nutrition students. Early screening, increasing awareness, and promoting healthy eating habits could be potential strategies to help treat and prevent the development of disorders or associated health conditions in nutrition as well as non-nutrition students.

## 1. Introduction

Eating disorders (ED) are defined by persistent disturbed eating behaviors that result in altered consumption or absorption of food and physical or psychological dysfunction [1]. Individuals who do not meet criteria for an eating disorder may engage in some forms of disordered eating behaviors (e.g., binge eating, restraint, emotional eating, disinhibition, strict dieting, and controlling body weight and shape through inappropriate compensatory behaviors), which are all risk factors for eating disorders [2,3]. Eating disorders commonly begin in adolescence and young adulthood, a life stage associated with stressful events such as leaving home for college [4,5]. Studies have shown the prevalence estimates of eating disorders among college-aged students have ranged from 8% to 20.5% [6,7,8]. Alarmingly, a significant portion of college-aged students who do display signs of EDs have neither been diagnosed, nor do they seek treatment [7]. Screening and early detection of disordered eating behaviors among college students seems to be a significant need.

Unhealthy eating practices such as dieting, fasting, vomiting, and abusing laxatives are factors that can affect the development of disordered eating behaviors [9]. As part of a multidisciplinary approach, nutrition counseling plays a significant role in the treatment of eating disorders and related complications [10,11]. With professional training in meal planning, healthy eating habits, and attitudes towards weight control at school, students majoring in nutrition might be at less risk for disordered eating behaviors than non-nutrition majors.

However, there exists a belief that nutrition students initiate their studies as motivation to deal with their own disordered eating behaviors. These behaviors could potentially pre-exist their nutrition studies but could also be the result of an overstressed concern with eating healthily during their coursework [12]. Globally, eating disorders are concerning in nutrition faculties. An international study revealed that 77% of nutrition professionals (e.g., professors, teachers, dietitians) from 14 countries felt eating disorders were a concern for nutrition students [13]. Several studies indeed suggested the prevalence of eating disorders in college students studying nutrition is higher than in students studying other majors [14,15,16]. In a study comparing eating behavior between Portuguese undergraduate nutrition students and students attending other courses, nutrition students presented higher restraint and binge eating than students from other courses [14]. In another study conducted in South Africa, a higher prevalence eating disorder risk in first-year nutrition and dietetic students was reported when compared to other non-nutrition related students (33.3% vs. 16.9%; *p* = 0.059) [17].

Other studies have reported different findings. In a study with data from 189 female Portuguese students aged 18–25 years old, there was no difference in risk of ED development between students majoring in nutrition and other health-related majors or non-health-related majors [18]. Another study collected data from 773 Turkish undergraduate students and reported that students studying Physical Education and Sports had a higher tendency for abnormal eating behavior and more concern for body shape than students studying Nutrition and Dietetics or Social Science [19]. In a cross-sectional comparison of nutrition students in Germany, nutrition students were inclined to restrict food intake for weight control; however, they did not display more disordered eating patterns compared to other students. Interestingly, they tended to adopt healthier food choices as they progressed through their nutrition studies [12].

Eating habits for college students are a topic of interest because the greatest increase in overweight and obesity occurs between the ages of 18–29 according to the Behavioral Risk Factor Surveillance System [20]. Additionally, data from the 1995 College Health Risk Behavior Survey, suggests diet and physical activity levels during college predispose this population to future health issues [20]. In a study of 764 college freshmen at an independent American university, 50% reported eating high-fat or fast food three or more times during the previous week [20]. These foods have been shown to promote addiction-like deficits in the brain reward function and may lead to overeating and obesity [21]. Food Addiction (FA) is a term that has been used to describe an abnormal pattern of compulsive consumption of certain types of foods such as foods high in sugar, fat, and/or salt [22,23,24,25,26,27]. Food addiction in humans is usually defined and measured by Yale Food Addiction Scale (YFAS), a 25 self-reported questions assessment tool, based on the Diagnostic and statistical Manual for Mental Disorders-IV (DSM-IV) criteria for substance dependence [23,28]. There are seven addiction symptoms for substance dependence according to DSM-IV. The diagnostic criteria for food addiction is three or more addiction symptoms are endorsed and criteria for a clinically significant impairment or distress is met [23,29]. According to a systematic review of 25 studies including 196,211 participants published in 2014, food addiction prevalence ranged from 7.8% to 25% for young adults (younger than 35 years old), with an average of 17% [29]. In populations with disordered eating, the average prevalence of food addiction rose to 57.6% [29]. Comparatively, for those without an eating disorder, food addiction was only 16.2% [29]. Food addiction prevalence was also twice as high in the overweight/obese population compared to those with a healthy BMI (24.9% and 11.1% respectively) [29]. In a study conducted with Chilean students aged 18–39, 11% met the criteria for food addiction when using the YFAS [30]. This study also observed a higher prevalence of food addiction (30%) among those who were classified as obese [30]. Yet another study showed prevalence of food addiction, according to YFAS criteria, to be 8.8% among junior college students [31]. There is evidence of large overlap between food addiction and binge eating. Among obese subjects with Binge Eating Disorder (BED), 56.8% of participants met the criteria for food addiction in one study [32] and 41.5% met the criteria in another study in a racially diverse population [33]. In another study, 100% of participants with current Bulimia Nervosa (BN) met FA criteria while 30% of participants with remitted BN did [34]. In addition, the co-occurrence of food addiction with eating disorders appears to be associated with worse clinical conditions and symptoms [35,36]. To date, no food addiction has been assessed among nutrition major college students yet. Regarding disordered eating relative to weight status, studies are inconsistent. In one study conducted on 548 college-aged women, no association was found between being overweight and having an eating disorder [37]. However, another study, focused on 715 female undergraduate students, did show an association between higher BMI and binge eating disorder as well as severity of binge eating symptoms [38].

The aim of the current study was to assess the prevalence of disordered eating behaviors among college students in a public university in the United States and to determine if there are any significant eating behavior differences between nutrition and non-nutrition major students. The food addiction prevalence was assessed and the difference between nutrition and non-nutrition major students was compared. Disordered eating behaviors and food addiction prevalence were also assessed in different weight status groups and academic years among college students.

## 2. Materials and Methods

### 2.1. Samples

**Figure 1:**
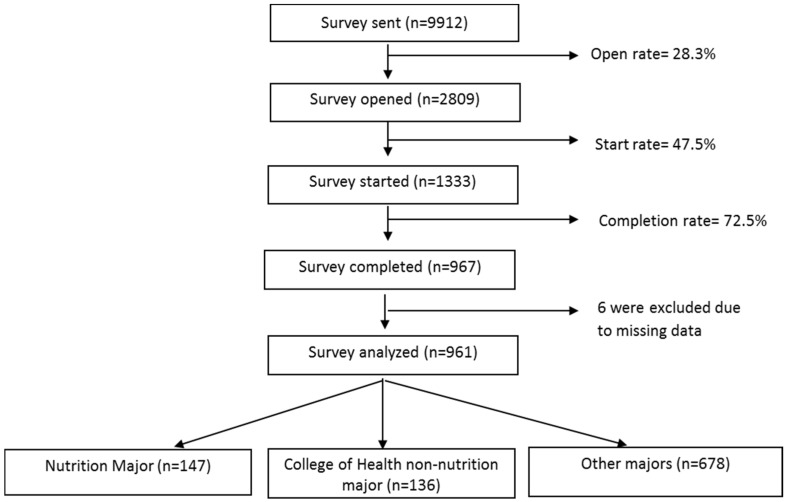
Study profile.

College students, ages 18–25, enrolled for spring 2014 term at a public university in Florida, United States were recruited through campus email. The email was sent out by the Academic Affair of the university via the Qualtrics email system to all college students aged 18–25 years old. If students agreed to participate, by clicking on the individual link in the email announcement, they will be instructed to complete informed consent and the survey through Qualtrics system. They were also instructed to print a copy of the consent document for their records. The survey link was active for two weeks from the date sent. To encourage nutrition major students to participate, faculty in the department of Nutrition and Dietetics were encouraged to reward bonus points for student participation. No other type of incentive was given to participants. Among 9912 college students to which questionnaires were sent, 967 respondents finished all questionnaires (response rate 9.8%). Six respondents were excluded from analysis due to incompleteness of questionnaires. Final respondents included in the analysis were 961 participants. Figure 1 provides details on response rate. 

Participants were classified as three groups: nutrition major (students majoring in Nutrition and Dietetics), non-nutrition health majors (students studying other majors in the College of Health, e.g., nursing, public health, health administration, and clinical and movement science), and other majors (all other majors outside College of Health). Previous studies have reported different tendency for abnormal eating behaviors or body concerns between health-related non-nutrition majors and nutrition major [19,39]. Therefore, current study distinguished these two groups and included three academic major groups. This study was approved by the university’s IRB committee on 27 February 2014 (UNF IRB number: 554391-2).

### 2.2. Measures

Four measures were used. The first measure assessed participants’ basic demographic characteristics, such as age, sex, height, weight, and race/ethnicity. Three other validated measures were used to assess specific disordered eating behaviors.

The Eating Attitude Test (EAT-26) is a widely used 26-item screening tool which assesses a broad range of symptoms of Anorexia Nervosa or Bulimia Nervosa. The EAT-26 alone does not yield a specific diagnosis for an eating disorder; however, it is a good first step to use the screening process. Study has shown it to be useful in assessing “eating disorder risk” [40]. EAT-26 contains three sub-scales: dieting (13 items), bulimia and food preoccupation (6 items), and oral control (7 items) [41]. The dieting scale relates to the avoidance of fattening foods and a preoccupation with thinness [42]. The bulimia and food preoccupation scale relates to food thoughts and bulimia [42]. The oral control scale relates to displaying self-control around food and the perceived pressures from others to eat more and gain weight [42]. The subscale scores are computed by summing all items assigned to that subscale. A total score is the sum of all three subscale scores. Higher EAT total scores indicate higher risk for eating disorders. An EAT score ≥ 20 is considered a positive EAT score, i.e., greater possibility of an eating disorder [43].

The Three Factor Eating Questionnaire (TFEQ-R18) is an 18 item self-assessment tool to measure three dimensions of eating behaviors (cognitive restraint, disinhibition or uncontrolled eating, and emotional eating) [44,45]. Cognitive restraint (six items) assesses the tendency to restrict food intake. Disinhibited eating (nine items) assesses the extent of loss of control over eating. Emotional eating (three items) assesses how emotions influence the perception of hunger and the urge to eat [44]. Strong association were obtained between the revised TFEQ scales and their corresponding factors [44]. Higher scores indicate higher cognitive restraint, uncontrolled eating, and a higher level of emotional eating. It was first developed for obesity research and is now widely used to investigate the links between restraint, eating disorders, and obesity [44,45]. It is reported that all three dimensions (cognitive restraint, disinhibition, and emotional eating) are associated with binge eating [46].

The Yale Food Addiction Scale (YFAS) is a 27-item tool that was developed in 2009 by modeling DSM-IV criteria for substance dependence and applying them to eating behaviors [23]. It measures seven food addiction symptoms: loss of control, persistent desire or unsuccessful repeated attempts to quit, large amount of time spent on substance, involvement in important activities given up, continued use despite problems, tolerance and withdrawal symptoms in eating behaviors, and attitudes about food preference [47]. Two scoring options are available: a symptom count score 0–7 and a dichotomous diagnostic score, assigned to individuals with three or more symptoms who also satisfy the clinical impairment criteria [29,47]. Both scoring methods were used in the current study.

### 2.3. Statistical Analysis

Descriptive statistics including frequencies (n; %), means, and standard deviations (SD) were used. BMI was computed from self-reported weight and height (kg/m^2^). Participants were classified as underweight (BMI < 18.5 kg/m^2^), normal weight (18.5 kg/m^2^ ≤ BMI < 25 kg/m^2^), overweight (25 kg/m^2^ ≤ BMI < 30 kg/m^2^), obese I (30 kg/m^2^ ≤ BMI < 35 kg/m^2^), or obese II (BMI ≥ 35 kg/m^2^). Height, weight, BMI, and continuous outcomes for eating behavior dimensions were compared using one-way ANOVA among different academic major categories (nutrition students, non-nutrition health major students, and other major students) followed by Tukey’s HSD Post Hoc tests. The group sizes were unequal, thus the harmonic mean of the group sizes was used. Category outcomes were compared using Chi-square tests among three different major categories. Respondents who were scored as high risk for eating disorders (EAT score ≥ 20) or who were diagnosed as food dependent (results from YFAS) were grouped into two BMI categories based on their weight status (underweight/normal vs. overweight/obese). Among-group difference and within-group difference between two weight status categories were analyzed using Chi-square tests. Those high risk respondents were also grouped into two academic categories based on their academic years (freshman or sophomore vs. junior or senior). Among- and within-group differences between two academic years categories were also analyzed using Chi-square tests. Co-occurrence of EAT score ≥ 20 and food addiction was also compared by weight status and by academic year category respectively using Chi-square tests. All analyses were performed using IBM SPSS Statistics for Windows, version 22.0. The significance level was set to α = 0.05.

## 3. Results

### 3.1. Respondent Characteristics

As shown in Table 1, among 961 respondents whose data were analyzed, 147 participants were from nutrition and dietetics programs; 136 participants were from other non-nutrition majors within Brooks College of Health; and the remaining 678 participants were from other majors at the university. Seven hundred and three respondents (72.7%) were women. Seven hundred and twenty-one respondents (74.8%) were white. Six hundred and thirty participants (65.6%) were at a healthy weight (BMI 18.5–25). Respondents were fairly evenly distributed among all school years with a slightly higher percentage being juniors (36.9%). Compared to students studying other academic majors, nutrition majors included more women (84.4% vs. 69.2%, *p* < 0.0001) and those with a lower BMI (22.99 vs. 24.17, *p* < 0.05).

### 3.2. Disordered Eating Behaviors and Food Addiction among Groups

As shown in Table 2, many participants engaged in disordered eating behaviors. EAT results indicated that overall 96 respondents (10.0%) were of a high level of concern regarding eating, body weight and body shape. There were no significant differences among the three academic major categories in all three EAT behavioral dimensions. The Dieting Scale result ranging from 5.81 to 6.33 indicates a relatively low level of dieting behaviors among participants. The Bulimia/Food Preoccupation Scale result ranging from 1.12 to 1.49 indicates relatively low level of bulimia-related behaviors and food thoughts. The Oral Control Scale result ranging from 1.49 to 1.92 indicates strong self-control around food by all participants.

TFEQ-R18 provided data about restrained eating, uncontrolled eating, and emotional eating. Specifically, respondents had moderate restrained eating (12.8 to 12.9; min–max: 6–24), moderate uncontrolled eating (18.5–18.8; min–max: 9–36), and moderate emotional eating scale scores (6.4–6.5; min–max: 3–12). There were no significant differences on all eating scales among different academic majors.

YFAS data reported that ninety-nine respondents (10.3%) met the criteria for food addiction—i.e., met symptom count and clinical significance standards. Eight dimensions of food dependence were assessed. The value of “loss of control” was lower in the current surveyed population than the norm value (8.6% vs. 21.7%). The values of “have tried unsuccessfully” and “tolerance” were higher in the current surveyed population compared to the norm value (89.7% vs. 71.3% and 25.4% vs. 13.5%). The values of all other dimensions were comparable to normal values that were surveyed among a healthy general population. When comparing nutrition majors with other groups, there were no significant differences in food dependence symptom count (nutrition major: 1.85 ± 1.35; non-nutrition health major: 1.96 ± 1.72; other major: 1.92 ± 1.55, *p* = 0.823), diagnosed “food dependence” (nutrition major: 9.5%, non-nutrition health major: 14%; other majors: 9.7%, *p* = 0.315), and nearly all eating behavior dimensions except for “withdrawal” with non-nutrition health majors demonstrating more withdrawal behaviors than other two groups (9.7% vs. 20.1% vs. 11.6%, *p* = 0.013).

### 3.3. High Risk Subgroup Analysis by Weight Status

To assess whether the risk for eating disorders varies by weight status group, all participants were divided into two combined weight status categories (underweight/normal and overweight/obese) since there were very few “underweight” and “obese” participants. As shown in Table 3, though not significantly different, overall the percentage of high risk participants was lower in underweight/normal weight participants than in overweight/obese participants (8.8% vs. 12.9%, *p* = 0.057). This trend was significantly found in the non-nutrition health major group (underweight/normal 5.6% vs. overweight/obese 25.9%, *p* = 0.0014). In nutrition and all other majors groups, the percentage of high risk participants was comparable between the underweight/normal weight category and the overweight/obese category (*p* > 0.05). There was no significant difference in high risk prevalence among the academic major groups in either weight category.

As shown in Table 3, overall prevalence of food dependence was significantly lower in the underweight/normal weight category than in the overweight/obese category (8.9% vs. 14.7%, *p* = 0.0087). This trend was consistently found significantly in non-nutrition health major group (10.3% vs. 28.6%, *p* = 0.015) and non-significantly in other two groups (other major: 8.3% vs. 13.0%, *p* = 0.0525; nutrition major: 9.1% vs. 11.8%, *p* = 0.646). There was no significant difference in prevalence of food dependence among the three academic major groups in either weight categories.

### 3.4. High Risk Subgroup Analysis by Academic Year

To assess whether the prevalence changes with progression through the program, all participants were divided into two academic year categories: Freshman/Sophomore and Junior/Senior. As shown in Table 4, overall the percentage of high risk participants (EAT ≥ 20) was comparable or slightly lower in early academic years than in late academic years (8.8% vs. 10.6%, *p* = 0.366). This pattern was consistently found in the non-nutrition health major group (8.3% vs. 11.1%, *p* = 0.582) and other majors group (8.4% vs. 11.1%, *p* = 0.247). Interestingly, in the nutrition major group, there was a slightly higher percentage of high risk of eating disorders participants in the freshman/sophomore year than in the junior/senior year (13.8% vs. 8.5%, *p* = 0.382). There was no statistically significant difference in total EAT score among the academic major groups in either academic year group.

Overall prevalence of food dependence was comparable in Freshman/Sophomore years to Junior/Senior years (9.5% vs. 11.0%, *p* = 0.479). This trend was found in the non-nutrition health major group (12.7% vs. 14.5%, *p* = 0.757) and other majors group (7.8% vs. 11.4%, *p* = 0.128). Again interestingly, in the nutrition major group, there was a slightly higher percentage of food dependence in freshman/sophomore year than in junior/senior year (17.9% vs. 7.7%, *p* = 0.102). There was no statistically significant difference in prevalence of food dependence among the three academic major groups in either academic year group.

### 3.5. Co-Occurrence of EAT Score ≥ 20 and Food Addiction Diagnosis by Weight Status

Table 5 presented co-occurrence of EAT Score ≥ 20 and food addiction diagnosis data by weight status. There were, overall, 43 respondents (4.5% or 4.6% depending on total valid respondents) who had co-occurrence of EAT Score ≥ 20 and FA diagnosis. This accounted for 44.8% of all participants who had an EAT score greater than 20 and 43.4% of all participants who met the criteria for FA diagnosis. Out of total respondents, 27 co-occurrences (4.0% or 4.1%) were either underweight or with normal weight and 16 co-occurrences (5.7% or 5.9%) were either overweight or obese (*p* > 0.05 between two weight categories). Out of participants who had an EAT score greater than 20, co-occurrence of EAT Score ≥ 20 and FA accounted for 45% in underweight or normal category and 44.4% in overweight or obese category (*p* > 0.05). Out of participants who met the criteria for FA diagnosis, co-occurrence accounted for 45.8% in the underweight or normal category and 40% in the overweight or obese category (*p* > 0.05).

### 3.6. Co-Occurrence of EAT Score ≥ 20 and Food Addiction Diagnosis by Academic Year

Table 6 presented co-occurrence of EAT Score ≥ 20 and food addiction diagnosis data by academic year category. There were overall 42 respondents (4.4% or 4.5% depending on total valid respondents) who had a co-occurrence of EAT Score ≥ 20 and FA diagnosis. This accounted for 44.2% of all participants who had an EAT score greater than 20 and 42.9% of all participants who met the criteria for FA diagnosis. Out of total respondents, 13 co-occurrences (3.6%) were either freshman or sophomore and 29 co-occurrences (4.9% or 5.0%) were either junior or senior students (*p* > 0.05 between two academic year categories). Out of participants who had EAT score greater than 20, co-occurrence of EAT Score ≥ 20 and FA accounted for 40.6% in freshman or sophomore category and 46% in junior or senior category (*p* > 0.05). Out of participants who met the criteria for FA diagnosis, co-occurrence accounted for 38.2% in freshman or sophomore category and 45.3% in junior or senior category (*p* > 0.05).

## 4. Discussion

This study assessed the prevalence of disordered eating behaviors and food addiction among nutrition versus non-nutrition major college students. To the best of our knowledge, this is the first study to assess the prevalence of food addiction, as assessed by the YFAS, in college students specifically majoring in nutrition. This is also one of the first studies to assess disordered eating behaviors in different weight status groups and academic years among college students. There are several important novel findings derived from this study.

There were 10% of participants at high risk of eating disorders (EAT ≥ 20) among surveyed respondents. This percentage was comparable to what was found in previous US studies, which ranged from 9% to 15% among college students including both genders [6,7,48,49]. Compared to other countries, this percentage is lower than what was reported from college students in a French study (20.5%) [8], in Pakistani medical students (22.75%) [50], in Malaysian university students (18.2%) [51], and students in a public university in Spain (17.6%) [52], but higher than in a Romanian study (7%) [42]. A much lower rate of disordered eating attitudes and behaviors was reported in Chinese college students (4.5%) [53]. The wide range of positive EAT scores may reflect the true difference in prevalence of eating disorders among college students in different geographic regions worldwide. The self-reported nature of the EAT questionnaire may also partially explain the wide range of prevalence. Current evidence does not indicate higher levels of disordered eating behaviors in more developed counties than in other countries because the prevalence among college students ranged from 9% to 20.5% in Western countries and 4.5%–23% in Eastern countries. Moreover, one study reported that Filipino students were 10.9 times more likely to have disordered eating behaviors than their American counterparts [54]. Almost all studies used EAT-26 as the instrument of measure. EAT-26 scores have shown high association with eating disorder symptoms and the questionnaire has shown high reliability [55]; therefore, differences were not caused by questionnaires used.

When comparing disordered eating behaviors among different majors, there was no difference among nutrition majors, health-related non-nutrition majors, and other majors. In addition, there were no differences among academic majors in either the EAT-26 subscales (i.e., dietary restraint, binge eating behavior, and oral control level) or the TFEQ-R18 subscales (i.e., cognitive restraint, loss of control, and emotional eating). These findings are in agreement with some studies [17,18,39], in which students in nutrition major were neither at higher risk of eating disorder nor differed in subscale behaviors, compared to students in other majors. This is inconsistent, however, with other studies, in which female nutrition and dietetics students had higher levels of disordered eating attitudes and behaviors (EAT-26 scores) compared to other first year program students [15], and nutrition students showed higher levels of dietary restraint than non-nutrition students [12,14]. In those studies, nutrition students might be at higher levels of food restriction in order to lose or maintain weight than in other majors [12,14]. However, the high restraint levels in first year college students might possibly have been counterbalanced by healthy approaches to weight control or healthier food choices and were not necessarily transformed to eating disorders in later program years [12]. In fact, two studies have reported that dietary restraint scores decreased in students of higher years in both nutrition and non-nutrition students [12,17].

Our study is the first study assessing food addiction, as assessed by YFAS, among college students and the difference between nutrition majors versus other majors. Overall, 10.3% of participants were identified as “food dependent”. This percentage is slightly lower than the norm score (11.6%), i.e., percentage of food dependence among the general public [23]. This number is also lower than some other reports. In one review article, the weighted mean prevalence of food addiction (FA) diagnosis was 19.9% for adults across 20 studies [29]. In adults younger than 35 years of age, the mean FA prevalence was 17.0% [29]. In our study, the number of FA symptoms was 1.91 ± 1.55, which is within the range of reported symptom counts from 20 studies (from 1.8 to 4.6) out of a possible total score of seven. In addition, the symptom count is comparable to the reported symptom count of non-clinical samples: 1.7 ± 0.4 [29]. Compared to other academic majors, nutrition major had a statistically indifferent prevalence of FA diagnosis and indifferent FA symptom count. This lack of significant differences in FA diagnosis and FA symptoms among three academic majors is consistent with our EAT-26 and TFEQ-R18 findings, which indicated that, in our sample population, the eating behaviors and attitudes, emotional control, and food addiction, etc., did not differ between nutrition and non-nutrition majors. Among all seven specific addiction symptoms, only withdrawal showed a significant difference with non-nutrition health majors having higher withdrawal symptoms than the other academic major categories. With current limited evidence, this may merit future investigation.

The present study reported that eating disorder high risk participants (EAT ≥ 20) were more prevalent among overweight/obese participants than underweight/normal participants in non-nutrition health major (Table 3, *p* < 0.005). In nutrition students and students studying other majors, however, risk prevalence was comparable between the overweight/obese group and the underweight/normal weight group (*p* > 0.05). According to a Romanian study, all surveyed medical students with high risk disordered eating behaviors (EAT ≥ 20) were underweight or normal weight [42]. That indicates that being overweight or obese did not increase the chances of having an eating disorder. It should be noted that there were only 70 total students surveyed and only 5 students had high EAT-26 scores. The small sample size of that study might reduce the generalization of their findings [42]. In another study, the percentage of students with EAT-26 ≥ 20 did not differ between BMI ≥ 25 and BMI < 25 in either African American or Caucasian college students [49]. This is consistent with one study of 4201 American college participants, in which researchers revealed that an EAT-26 score of ≥20 was not associated with weight status [48]. A similar percentage of students with disordered eating behaviors was reported in a normal weight group and an overweight/obese group (EAT ≥ 20: 15.0% in normal weight vs. 15.3% in overweight/obese). An EAT-26 score of ≥20 has been suggestive of anorexia nervosa and bulimia nervosa [56]. For nutrition and all other major students, normal weight status does not necessarily indicate low risk of disordered eating behavior. High EAT ≥ 20 while being normal weight might suggest dieting or risk of anorexia nervosa. Alternatively, more EAT ≥ 20 participants in the overweight/obese group than the underweight/normal weight group in non-nutrition health majors may indicate more binge eaters in this group. One study has reported more college students with an EAT-26 score ≥ 11 in overweight/obese group than normal weight group [48]. EAT-26 score of ≥11 has been associated with a high risk of binge eating disorder [57]. Thus, weight status might be an indicator of binge eating, or binge eating disorder, among college students. Screening and treating weight problems may facilitate the treatment of disordered eating behaviors for these students.

In our study, food dependence was more prevalent in overweight/obese participants than in underweight/normal weight participants overall (*p* < 0.01) and for non-nutrition health major (*p* < 0.05). A non-significant but similar pattern was also found in nutrition and all other students. There was evidence from one study that higher prevalence of food addiction is found among overweight and obese adults (25%) [58]. Increased food addiction symptomology was also suggested to relate to less short-term weight loss (seven weeks) [47]. The present study together with previous literature suggest that “food addiction” might be a valid phenotype of obesity in the college student age population, including nutrition students. Reducing weight might help relieve the food addiction symptoms for students.

Though non-statistically significant, our study reported that less nutrition students engaged in disordered eating behaviors (EAT-26 ≥ 20) in higher academic years compared to lower academic years. This is consistent with some other reports. A South African study reported that there was a non-significantly lower prevalence of disordered eating in junior/senior dietetic students compared to freshman dietetic students (18.4% vs. 33.3%, *p* = 0.151) [17]. In the same study, there was an observed trend of lower levels of dietary restraint and disinhibition in later years of study than freshman year [17]. In another study, more advanced nutrition students showed healthier food choices (freshman vs. seventh semester and above), whereas the corresponding controls showed slightly greater unhealthy food choices [12]. However, with relatively small sample size and statistical non-significance in the present study, we cannot make a definitive conclusion.

Similarly, as students stayed in the program longer, there was a non-significant lower percentage of nutrition students being classified as “food dependent”. In the other two major categories, the percentage showed little change across years. Our study is the first study to report food addiction changes in different years of nutrition students and would benefit from further investigation with larger sample sizes and in other populations.

In the current study, 43% to 45% of the high risk participants (EAT ≥ 20 or FA) had co-occurrence of both eating disorder risk and food addiction risk. In a previous large non-clinical sample, 47.1% of binge eating disorder participants endorsed “food addiction” while 83.6% of bulimia nervosa participants met “food addiction” threshold [35]. It was reported that co-occurrence of food addiction and binge eating or binge eating related eating disorders (BED and BN) was associated with more severe psychopathology (i.e., anxiety and depressive symptoms) and clinical symptoms (i.e., time spent dieting, subjective binge eating episode, disordered eating attitude) [35,36]. When screening and treating for eating disorders, identifying people presenting food addiction may be important for clinical treatment. The co-occurrence was not associated with weight status or academic year status in our participants. This may be contributed by the relatively small sample of people who had co-occurrence of disordered eating behavior and food addiction. More research is warranted regarding this.

Concerns of high eating disorder prevalence in nutrition students have been expressed widely by nutrition educators of the world [13,59]. The current study did not find a higher prevalence of disordered eating behavior among nutrition majors than other majors; however, overall 10% of participants reported disordered eating behaviors indicating the need of increasing awareness of eating disorders among college students. In a study from 14 counties, 48% of nutrition faculty members thought screening for eating disorders in nutrition students would be a good idea [13]. The current study suggests that screening for eating disorders campus-wide might be a necessary prevention approach for eating disorders.

This study has a couple of limitations. First, there might be response bias. Because the data is self-reported, there might be a potential for socially desirable responding from participants. Students may be inclined to underreport symptoms. Second, the measures did not include assessments of general psychopathology (e.g., depressive and anxiety symptoms). These psychological behaviors are strongly related with food addiction and disordered eating behaviors [32,60]. Third, the measures in current study were chosen based on similar research studies using the same measures. Though to our best knowledge, there is not a “gold standard” with respect to assessing disordered eating behaviors, some other measures—e.g., Eating Disorder Examination Questionnaire (EDE-Q)—might be considered for a more comprehensive assessment in the future. Fourth, this study was a cross-sectional survey. It would be beneficial to have a prospective cohort study that follows the same cohort from first year to graduation. Fifth, the current study only included undergraduate students of nutrition majors. It would be interesting to observe any difference between undergraduate vs. graduate nutrition students.

## 5. Conclusions

Our study demonstrated that disordered eating behaviors are a concern facing both nutrition and non-nutrition students. Screening among college students campus-wide, increasing awareness of eating disorders, promoting healthy eating habits, and encouraging overweight/obese students to lose weight could be potential strategies to help reduce the development of eating disorders among all college students.

## Figures and Tables

**Table 1:** Baseline characteristics *.

	All (*n* = 961)	Nutrition (*n* = 147)	COH ^#^ Non-Nutrition (*n* = 136)	Other Majors (*n* = 678)	*p*-Value
Sex					
Male	262 (27.2%)	23 (15.6%)	29 (21.5%)	209 (30.8%)	<0.0001
Female	699 (72.7%)	124 (84.4%)	106 (78.5%)	469 (69.2%)	
Height (inches) **	66.32 ± 3.71	65.44 ± 3.16 ^a^	66.23 ± 3.51 ^a,b^	66.53 ± 3.83 ^b^	<0.01
Weight (lbs) **	150.10 ± 37.08	140.38 ± 27.78 ^a^	147.16 ± 35.37 ^a,b^	152.79 ± 38.77 ^b^	0.001
BMI **	23.89 ± 5.16	22.99 ± 4.03 ^a^	23.43 ± 4.40 ^a,b^	24.17 ± 5.49 ^b^	<0.05
BMI category					
Underweight < 18.5	51 (5.3%)	6 (4.1%)	7 (5.2%)	38 (5.6%)	NS
Normal 18.5–25	630 (65.6%)	105 (71.9%)	100 (74.1%)	422 (62.4%)	
Overweight 25–30	186 (19.4%)	28 (19.2%)	17 (12.6%)	140 (20.7%)	
Obese I 30–35	77 (8.0%)	6 (4.1%)	10 (7.4%)	61 (9.0%)	
Obese II > 35	17 (1.8%)	1 (0.7%)	1 (0.7%)	15 (2.2%)	
Ethnicity					
White	717 (74.8%)	107 (74.1%)	100 (73.5%)	510 (75.4%)	NS
African American	60 (6.3%)	9 (6.1%)	12 (8.8%)	39 (5.8%)	
Hispanic	78 (8.2%)	9 (6.1%)	13 (9.6%)	56 (8.3%)	
Asian/Pacific Islander	57 (6.0%)	14 (9.5%)	6 (4.4%)	37 (5.5%)	
Others	45 (4.7%)	6 (4.1%)	5 (3.7%)	34 (5.0%)	
Academic Year					
Freshman	179 (18.6%)	12 (8.2%)	36 (26.7%)	131 (19.4%)	<0.0001
Sophomore	185 (19.3%)	17 (11.6%)	36 (26.7%)	132 (19.5%)	
Junior	353 (36.9%)	69 (46.9%)	41 (30.4%)	243 (35.9%)	
Senior	242 (25.2%)	49 (33.3%)	22 (16.3%)	171 (25.3%)	

* Data were presented as mean ± SD or *n* (%). NS: non-significance, *p* > 0.05; ^#^ COH: College of Health; ** Different letters indicate difference among different academic majors.

**Table 2:** Disordered eating behaviors *.

			**All (*n* = 961)**	**Nutrition (*n* = 147)**	**COH ^#^ Non-Nutrition (*n* = 136)**	**Other Majors (*n* = 678)**	***p*-Value**
	**Theoretical Range**	**Min–Max**	**Value**				
EAT-26							
Dieting scale			6.21 ± 6.56	6.33 ± 6.14	5.81 ± 6.28	6.26 ± 6.70	NS
Bulimia/Food preoccupation			1.31 ± 2.61	1.49 ± 2.44	1.12 ± 2.33	1.31 ± 2.70	NS
Oral control scale			1.81 ± 2.28	1.49 ± 1.47	1.57 ± 1.89	1.92 ± 2.47	NS
Total score			9.32 ± 9.34	9.31 ± 7.83	8.49 ± 8.72	9.49 ± 9.76	NS
EAT score ≥ 20 (%)			96 (10.0%)	14 (9.5%)	14 (10.3%)	68 (10.0%)	NS
TFEQ-R18							
Cognitive restraint	6–24	6–24	12.9 ± 3.8	12.9 ± 3.6	12.8 ± 3.1	12.9 ± 4.0	NS
Uncontrolled eating	9–36	9–36	18.7 ± 5.6	18.8 ± 5.5	18.5 ± 5.1	18.7 ± 5.6	NS
Emotional eating	3–12	3–12	6.4 ± 2.6	6.5 ± 2.5	6.5 ± 2.6	6.4 ± 2.7	NS
			**All (*n* = 942)**	**Nutrition (*n* = 145)**	**COH Non-Nutrition (*n* = 134)**	**Other Majors (*n* = 663)**	***p*-Value**
	**Theoretical Range**	**Min–Max**	**Value**				
YFAS							
Symptom count	0–7	0–7	1.91 ± 1.55	1.85 ± 1.35	1.96 ± 1.72	1.92 ± 1.55	NS
“Food dependence” diagnosis	11.6%		99 (10.3%)	14 (9.5%)	19 (14%)	66 (9.7%)	NS
Loss of control	21.7%		81 (8.6%)	12 (8.3%)	11 (8.2%)	58 (8.7%)	NS
Have tried unsuccessfully	71.3%		845 (89.7%)	130 (89.7%)	122 (91.0%)	593 (89.4%)	NS
Large amount of time spent	24.0%		177 (18.8%)	29 (20.0%)	29 (21.6%)	119 (17.9%)	NS
Important activities given up	10.3%		128 (13.6%)	16 (11.0%)	20 (14.9%)	92 (13.9%)	NS
Continued despite problems	28.3%		251 (26.6%)	36 (24.8%)	26 (19.4%)	189 (28.5%)	NS
Tolerance	13.5%		239 (25.4%)	35 (24.1%)	32 (23.9%)	172 (25.9%)	NS
Withdrawal **	16.3%		118 (12.5%)	14 (9.7%) ^a^	27 (20.1%) ^b^	77 (11.6%) ^a^	<0.05
Clinical significance	14%		129 (13.7%)	17 (11.7%)	24 (17.9%)	88 (13.3%)	NS

* Data were presented as mean ± SD or *n* (%). NS: non-significance, *p* > 0.05; ^#^ COH: College of Health; ** Different letters indicate difference among different academic majors.

**Table 3:** EAT Score ≥ 20 and food addiction diagnosis by weight status *.

	**All (*n* = 957)**	**Nutrition (*n* = 145)**	**COH ^#^ Non-Nutrition (*n* = 134)**	**Other Majors (*n* = 678)**	***p*-Value ^1^**
Total					
Underweight/Normal	678	111	107	460	
Overweight/Obese	279	34	27	218	
EAT ≥ 20					
Underweight/Normal	60 (8.8%)	11 (9.9%)	6 (5.6%)	43 (9.3%)	NS
Overweight/Obese	36 (12.9%)	2 (5.9%)	7 (25.9%)	27 (12.4%)	NS
*p*-value ^2^	NS	NS	<0.005	NS	
	**All (*n* = 939)**	**Nutrition (*n* = 144)**	**COH Non-Nutrition (*n* = 133)**	**Other Majors (*n* = 662)**	***p*-Value ^1^**
Total					
Underweight/Normal	666	110	105	451	
Overweight/Obese	273	34	28	211	
Food addiction (YFAS)					
Underweight/Normal	59 (8.9%)	10 (9.1%)	11 (10.5%)	38 (8.4%)	NS
Overweight/Obese	40 (14.7%)	4 (11.8%)	8 (28.6%)	28 (13.3%)	NS
*p*-value ^2^	<0.01	NS	<0.05	NS	

* Data were presented as *n* (%). NS: non-significance, *p* > 0.05; ^1^ *p*-value between three academic majors; ^2^ *p*-value within each academic major between two weight status categories; ^#^ COH: College of Health.

**Table 4:** EAT Score ≥ 20 and food addiction diagnosis by academic major year category *.

	**All (*n* = 959)**	**Nutrition (*n* = 147)**	**COH ^#^ Non-Nutrition (*n* = 135)**	**Other Majors (*n* = 677)**	***p*-Value ^1^**
Total					
Freshman/Sophomore	364	29	72	263	
Junior/Senior	595	118	63	414	
EAT ≥ 20					
Freshman/Sophomore	32 (8.8%)	4 (13.8%)	6 (8.3%)	22 (8.4%)	NS
Junior/Senior	63 (10.6%)	10 (8.5%)	7 (11.1%)	46 (11.1%)	NS
*p*-value ^2^	NS	NS	NS	NS	
	**All (*n* = 940)**	**Nutrition (*n* = 145)**	**COH Non-Nutrition (*n* = 133)**	**Other Majors (*n* = 662)**	***p*-Value ^1^**
Total					
Freshman/Sophomore	357	28	71	258	
Junior/Senior	583	117	62	404	
Food addiction (YFAS)					
Freshman/Sophomore	34 (9.5%)	5 (17.9%)	9 (12.7%)	20 (7.8%)	NS
Junior/Senior	64 (11.0%)	9 (7.7%)	9 (14.5%)	46 (11.4%)	NS
*p*-value ^2^	NS	NS	NS	NS	

* Data were presented as *n* (%). NS: non-significance, *p* > 0.05; ^1^ *p*-value between three academic majors; ^2^ *p*-value within each academic major between two academic major year categories; ^#^ COH: College of Health.

**Table 5:** Co-occurrence of EAT Score ≥ 20 and food addiction (FA) diagnosis by weight status.

	Total	Underweight/Normal	Overweight/Obese	*p*-Value ^1^
EAT ≥ 20 and FA	43	27	16	
Total	957	678	279	
EAT ≥ 20	96	60	36	
% of total	4.5%	4.0%	5.7%	NS
% of all EAT ≥ 20	44.8%	45%	44.4%	NS
EAT ≥ 20 and FA	43	27	16	
Total	939	666	273	
FA	99	59	40	
% of total	4.6%	4.1%	5.9%	NS
% of all FA	43.4%	45.8%	40%	NS

**^1^** *p*-value between two weight status categories. NS: non-significance, *p* > 0.05.

**Table 6:** Co-occurrence of EAT Score ≥ 20 and food addiction (FA) diagnosis by academic year.

	Total	Freshman/Sophomore	Junior/Senior	*p*-Value ^1^
EAT ≥ 20 and FA	42	13	29	
Total	959	364	595	
EAT ≥ 20	95	32	63	
% of total	4.4%	3.6%	4.9%	NS
% of all EAT ≥ 20	44.2%	40.6%	46%	NS
EAT ≥ 20 and FA	42	13	29	
Total	940	357	583	
FA	98	34	64	
% of total	4.5%	3.6%	5.0%	NS
% of all FA	42.9%	38.2%	45.3%	NS

**^1^** *p*-value between two academic major year categories. NS: non-significance, *p* > 0.05.
